# Multi-Particle-Collision Simulation of Heat Transfer in Low-Dimensional Fluids

**DOI:** 10.3390/e27050455

**Published:** 2025-04-24

**Authors:** Rongxiang Luo, Stefano Lepri

**Affiliations:** 1 Department of Physics, Fuzhou University, Fuzhou 350108, China; 2 Fujian Science and Technology Innovation Laboratory for Optoelectronic Information of China, Fuzhou 350108, China; 3 Consiglio Nazionale delle Ricerche, Istituto dei Sistemi Complessi, Via Madonna del Piano 10, I-50019 Sesto Fiorentino, Italy; stefano.lepri@isc.cnr.it; 4 Istituto Nazionale di Fisica Nucleare, Sezione di Firenze, Via G. Sansone 1, I-50019 Sesto Fiorentino, Italy

**Keywords:** low-dimensional fluids, MPC method, anomalous transport

## Abstract

The simulation of the transport properties of confined, low-dimensional fluids can be performed efficiently by means of multi-particle collision (MPC) dynamics with suitable thermal-wall boundary conditions. We illustrate the effectiveness of the method by studying the dimensionality effects and size-dependence of thermal conduction, since these properties are of crucial importance for understanding heat transfer at the micro–nanoscale. We provide a sound numerical evidence that the simple MPC fluid displays the features previously predicted from hydrodynamics of lattice systems: (1) in 1D, the thermal conductivity κ diverges with the system size *L* as $\kappa ∼L^{1/3}$ and its total heat current autocorrelation function C(t) decays with the time *t* as $C\left(t\right)∼t^{-2/3}$; (2) in 2D, κ diverges with *L* as κ∼ln(L) and its C(t) decays with *t* as $C\left(t\right)∼t^{-1}$; (3) in 3D, its κ is independent with *L* and its C(t) decays with *t* as $C\left(t\right)∼t^{-3/2}$. For weak interaction (the nearly integrable case) in 1D and 2D, there exists an intermediate regime of sizes where kinetic effects dominate and transport is diffusive before crossing over to the expected anomalous regime. The crossover can be studied by decomposing the heat current in two contributions, which allows for a very accurate test of the predictions. In addition, we also show that, upon increasing the aspect ratio of the system, there exists a dimensional crossover from 2D or 3D dimensional behavior to the 1D one. Finally, we show that an applied magnetic field renders the transport normal, indicating that pseudomomentum conservation is not sufficient for the anomalous heat conduction behavior to occur.

## 1. Introduction

Simulation is often the only viable tool to study many-particle systems driven away from equilibrium, especially where external mechanical and thermal forces are strong. Molecular dynamics is the most natural approach, but may be computationally expensive. Since one is often interested in large-scale properties, the details of microscopic interactions may not be essential, since only the basic conservation laws should matter. It is thus sensible to look for methods based on stochastic processes that may effectively account for molecular interactions. One popular approach is the multi-particle collision (MPC) dynamics, a mesoscale description where individual particles undergo stochastic collisions, rather than genuine Newtonian forces. The implementation was originally proposed by Malevanets and Kapral [1,2] and consists of two distinct stages: a free streaming and a collision one. Collisions occur at fixed discrete time intervals, and space is discretized into cells that define the collision range. The method captures both thermal fluctuations and hydrodynamic interactions.

The MPC dynamics is a useful tool to investigate concrete systems and has indeed been used in the simulation of a variety of problems, like polymers in the solution [1], colloidal fluids [3], plasmas and even dense stellar systems [4] etc. Besides its computational convenience, it is also a useful approach to address fundamental problems in statistical physics [5,6], and in particular, the effect of external sources.

Understanding heat transfer mechanisms in complex fluids, such as nanofluids and ionic liquids, is fundamental and has far-reaching implications across scientific and engineering disciplines [7,8,9,10]. In this work, we focus on the application of the MPC method to study heat transfer in a simple fluid both at equilibrium and in the presence of external reservoirs. In particular, we analyze the dimensionality effects on thermal transport in mesoscopic and confined fluids. Energy transport in low-dimensional systems has been thoroughly studied in recent decades and is crucial for achieving an understanding of macroscopic irreversible heat transfer on the nanoscale [11,12,13]. Also, it serves as a theoretical foundation for thermal energy control and management [14,15,16]. This is even more relevant at the nano- and microscale, where novel effects caused by reduced dimensionality, disorder, and nano-structuring affect the natural and artificial materials [17]. One remarkable property is that, in low-dimensional many-particle systems, energy propagates super-diffusively, implying a breakdown of classical Fourier’s law. This has been well studied, mostly in lattice systems but much less in fluids. We will show that the MPC dynamics is particularly effective and allows for a very accurate test of existing theories of anomalous transport.

This paper is organized as follows. In Section 2 we recall the basic definitions of the MPC dynamics and the thermal-wall method used to enforce the interaction with external heat baths. We then present the results of both the equilibrium and non-equilibrium for the one- (1D), two- (2D), and three-dimensional (3D) mesoscopic fluids in Section 3, Section 4 and Section 5, respectively. In Section 6, we illustrate how, upon changing the aspect ratio of the simulation box, one can observe crossover behavior in size dependence of the transport coefficient. In Section 7, we briefly discuss the case 2D fluids with magnetic field to clarify that pseudomomentum conservation is not the necessary condition for the anomalous heat conduction behavior.

## 2. Mesoscopic Fluid and Thermal Walls

**Figure 1:**
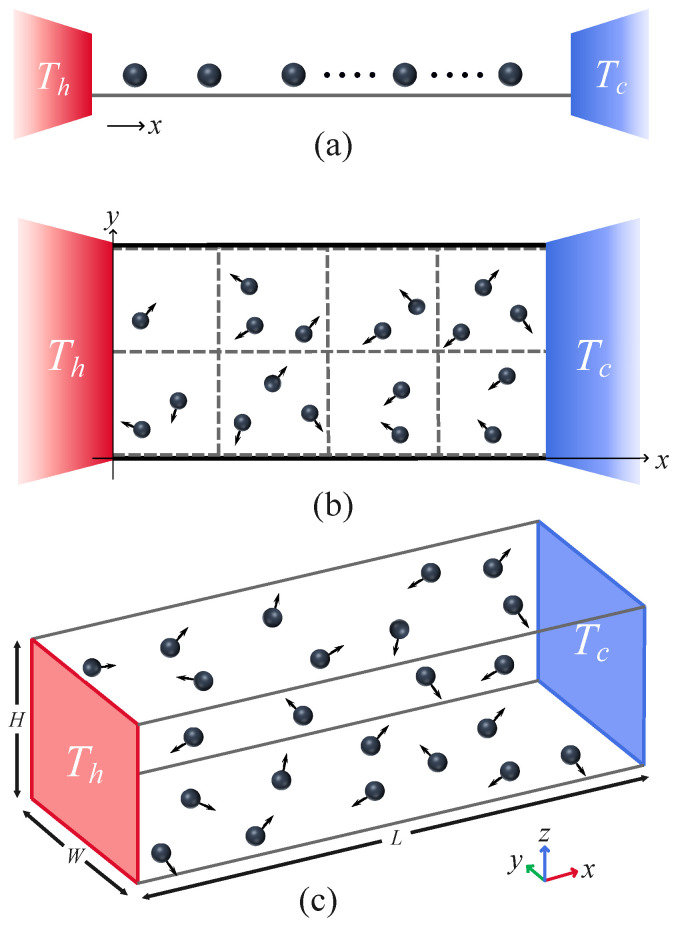
(Color online) Schematic illustration of 1D (**a**), 2D (**b**) and 3D (**c**) fluid of interacting particles in a system volume described by the MPC dynamics. The system is coupled at its left and right ends to one of two heat baths at fixed temperatures of $T_{h}$ and $T_{c}$ (see text for more details).

The 1D, 2D, and 3D mesoscopic fluid models we consider in this study are shown in Figure 1. The fluid consists of *N* interacting point particles with equal mass *m*, confined in a system volume. As is shown, in a 1D system, its volume has a length of *L* in the *x* direction; in the 2D system, its volume also has a width of *W* in the *y* direction; in 3D system, its volume has an additional height of *H* in the *z* direction.

At the boundaries x=0 and x=L, the particles interact with a heat bath of temperature $T_{h}$ or $T_{c}$; the heat baths are modeled as thermal walls [18,19]. When a particle crosses the x=0 (or x=L) boundary, it is reflected back with a new random velocity ($v_{x}$, $v_{y}$, and $v_{z}$ in the *x*, *y*, and *z* directions) assigned by sampling from a given distribution [18,19]:

(1) $$\begin{array}{cc} f\left(v_{x}\right)= & \frac{m|v_{x}|}{k_{B}T_{\alpha }}\exp\left(-\frac{mv_{x}^{2}}{2k_{B}T_{\alpha }}\right),\\ f(v_{y,z})= & \sqrt{\frac{m}{2\pi k_{B}T_{\alpha }}}\exp\left(-\frac{mv_{y,z}^{2}}{2k_{B}T_{\alpha }}\right), \end{array}$$

where $T_{\alpha }$ (α=h,c) is the temperature of the heat bath in dimensionless units and $k_{B}$ is the Boltzmann constant. The particles are subject to periodic boundary conditions in the *y* and *z* directions. We point out that the numerical results also apply to fixed boundary conditions since, in both cases, $v_{y,z}>0$ and $v_{y,z}<0$ with equal probability p=0.5.

Interaction among the particles, as prescribed by the MPC method [1,3,20], amounts to first partition the simulation box into many cells of linear size *a* (as is shown in Figure 1b for 2D case). The dynamics evolves in discrete time steps, each step consisting of a free propagation during a time interval τ followed by an instantaneous collision event. During propagation, the velocity $v_{i}$ of a particle is unchanged, and its position is updated as

(2) $$r_{i}\to r_{i}+\tau v_{i}.$$

For all particles in a given cell, their velocities are updated according to the following collision rules:

- In the 1D case, the velocity of the *i*th particle in the *j*th cell is changed according to the update rule
  (3) $$v_{i}\to A_{j}\omega_{i}+B_{j},$$
  where $\omega_{i}$ is randomly sampled by a thermal distribution at the cell kinetic temperature $T_{j}$, while $A_{j}$ and $B_{j}$ are cell-dependent parameters, determined by the condition of total momentum and total energy conservation in the cell [21].
- In the 2D case, all particles found in the same cell are rotated around the *z* axis, with respect to their center of mass velocity $V_{c.m.}$ by two angles, θ or −θ, randomly chosen with equal probability. The velocity of the *i*th particle in a cell is thus updated as
  (4) $$v_{i}\to V_{c.m.}+{\hat{R}}^{\pm \theta }\left(v_{i}-V_{c.m.}\right),$$
  where ${\hat{R}}^{\pm \theta }$ are the rotation operators by the angle ±θ.
- In the 3D case, the velocity of the *i*th particle in a cell is updated as in 2D case, with the difference that the rotation axis is also randomly selected.

The resulting motion conserves the total momentum and energy of the fluid. Note that the angle θ=π/2 for 2D and 3D cases corresponds to the most efficient mixing of the particle momenta. Also note that the probability of collision between particles increases as τ decreases. Thus, the time interval τ can be changed to tune the strength of the interactions that, in turn, will affect the transport properties.

In the non-equilibrium setup, we set $T_{\alpha }$ to be slightly biased from the nominal temperature *T*, i.e., $T_{h,c}=T\pm \Delta T/2$, to investigate the dependence of the thermal conductivity κ on the system length *L*. In our simulations, each particle is initially given by a random position’s uniform distribution and a random velocity generated from the Maxwellian distribution at the temperature *T*. After the system reaches the steady state, we compute the thermal current *J* that crosses the system according to its definition (i.e., the average energy exchanged in the unit time and unit area between particles and heat bath, see [22] for numerical details of computing non-equilibrium averages of the thermal current) and taking it into Fourier’s law, $\kappa =JL/(T_{h}-T_{c})$, to compute κ. We thus examine the dependence of κ on *L*, to assess whether the heat conduction behavior of the system is anomalous or normal.

In the integrable case (i.e., when τ=∞, each particle maintains unchanged velocity as it crosses the system from one heat bath to the other), by summarizing the results obtained before [23,24,25], we can obtain an analytical expression for the thermal conductivity:

(5) $$\kappa =L\left(d+1\right)\sqrt{\frac{\rho^{2}k_{B}^{3}}{2\pi m}}/\left(\frac{1}{\sqrt{T_{h}}}+\frac{1}{\sqrt{T_{c}}}\right).$$

where *d* is the spatial dimension and ρ is the particle density. It is shown that in the integrable case, transport is ballistic and the thermal conductivity is a linear function of the system length. This analytical result will be used to compare with our simulations as a numerical verification.

In the non-equilibrium setting, the difference between normal and abnormal heat conduction behaviors can be also appreciated upon examining the steady-state kinetic temperature profiles T(x). In our simulations, T(x) is measured as described in [24]. For systems with normal heat conduction, T(x) is determined by solving the stationary heat equation assuming that the thermal conductivity is proportional to $\sqrt{T}$ as prescribed by standard kinetic theory, yielding [26]

(6) $$T\left(x\right)={\left[T_{h}^{3/2}\left(1-\frac{x}{L}\right)+T_{c}^{3/2}\frac{x}{L}\right]}^{2/3}.$$

this prediction will be used to compare with our simulation results for normal heat conduction. On the other hand, for systems with abnormal heat conduction, T(x) is expected to be qualitatively different, being a solution of a fractional diffusion equation as demonstrated in several examples [27,28]. A typical feature is that the temperature profile is concave upwards in part of the system and concave downwards elsewhere, and this is true even for small temperature differences [29,30]. This will also be used to check our numerical simulations for abnormal heat conduction.

To check the results obtained in the non-equilibrium modeling, we will further turn to the comparison with linear-response results obtained in equilibrium modeling. Based on the celebrated Green–Kubo formula, which relates transport coefficients to the current time-correlation functions C(t), the thermal conductivity can be expressed as [11,12,31]

(7) $$\kappa_{\mathrm{GK}}=\frac{\rho }{k_{B}T^{2}}\lim_{\tau_{\mathrm{tr}}\to \infty }\lim_{N\to \infty }\frac{1}{N}\int_{0}^{\tau_{\mathrm{tr}}}C\left(t\right)dt.$$

In this formula, $C\left(t\right)\equiv \langle J(0)J(t)\rangle$ and $J\equiv \frac{1}{2}\Sigma_{i}^{N}v_{i}^{2}v_{x,i}$ represents the total heat current along the *x* coordinate in the equilibrium state. In the simulations, we consider an isolated fluid with periodic boundary conditions also in the *x* direction. The initial condition is randomly assigned with the constraints that the total momentum is zero and the total energy corresponds to *T*. The system is then evolved and after the equilibrium state is attained, we compute C(t) and the integral in Equation (7). Usually, the integral is truncated up to $\tau_{\mathrm{tr}}=L/v_{s}$ ($v_{s}$ is the sound speed) [11,12]. This results in the superdiffusive heat transport $\kappa_{\mathrm{GK}}∼L^{1-\lambda }$ as long as C(t) decays as $∼t^{-\lambda }$ with λ<1.

In order to compare κ and $\kappa_{\mathrm{GK}}$ more accurately, we can resort to the spatiotemporal correlation function of local heat currents to compute the sound speed $v_{s}$ of the system in Equation (7). The spatiotemporal correlation function of local heat currents is defined as [32,33,34]

(8) $$C\left(x,t\right)\equiv \langle J^{loc}\left(0,0\right)J^{loc}\left(x,t\right)\rangle .$$

Numerically, we compute $C\left(x,t\right)$ as performed in [35]: the system is divided into $\frac{L}{b}$ bins in space of equal width b=0.2; the local heat current in the *k*th bin and at time *t* is defined as $J^{loc}\left(x,t\right)\equiv \sum_{i}\frac{1}{2}m_{i}v_{i}^{2}v_{x,i}$, where x≡kb and the summation is taken over all particles that reside in the *k*th bin. It is found that $C\left(x,t\right)$ features a pair of pulses moving away from x=0 at the sound speed [36], which are recognized to be the hydrodynamic sound modes. Their moving speeds of the two pulses allows us to estimate the sound of speed $v_{s}$ within the fluid.

As for the choice of parameters, in both non-equilibrium and equilibrium settings, we set T=1, ΔT=0.2, $m=k_{B}=a=1$, θ=π/2, and ρ=5 throughout the paper. In addition, for all data points shown in the figures, the errors are ≤1%. We note that, when the mean free path *ℓ* that particles stream between rotations is much smaller than *a*, that is $l=\tau \sqrt{\frac{k_{B}T}{m}}\ll a$, randomly shifted cells for the collisions should be taken to guarantee the Galilean invariance of the stochastic rotation dynamics, so as to avoid artificial artifacts [37]. We here point out that, in this work, randomly shifted cells have not been used for collisions, because most of our simulations do not fall into the case where l≪a.

## 3. One-Dimensional Fluid

### 3.1. Non-Equilibrium Results

To start, let us discuss the dependence of the thermal conductivity κ on the system length *L* with different interaction strengths. Here, the time interval τ between successive collisions will be used to tune the strength of the interactions. The particle mean free path *ℓ*, in an uniform system, is proportional to the thermal velocity of the fluid $v_{T}=\sqrt{T}$ and the time of the MPC move, namely $l∼v_{T}\tau$.

**Figure 2:**
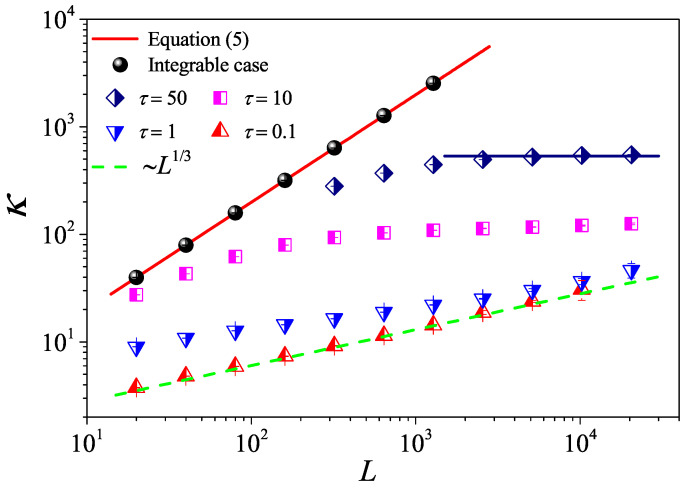
(Color online) The thermal conductivity κ as a function of the system length *L* for the 1D fluid system with different τ values. The symbols are for the numerical results, and for reference, the green dashed line indicates the divergence with *L* as $∼L^{1/3}$. For τ=50, the horizontal line denotes the saturation value of $\kappa_{\mathrm{GK}}$ obtained by Equation (7), where the integration is up a time $L/v_{s}$ with $v_{s}$ measured in Figure 5 bellow.

To check the results and provide a numerical example, we first quantify the non-interacting system τ=∞. In Figure 2, we report that κ of the 1D case (Equation (5)) with a red line is compared with our simulations (black cycles). It can be seen that they agree very well with each other. These simulations clearly strongly support our analysis.

We then turn to the interacting systems with τ<∞: It can be seen in Figure 2 that, for weak interactions (τ=50,10), κ tends to saturate and becomes constant as *L* is increased, following the Fourier law. However, it can also be seen that for the strong interactions (τ=1,0.1), κ is no longer constant but diverges with *L*. In particular, for τ=0.1, κ eventually approaches the scaling $\kappa ∼L^{1/3}$ like that predicted in 1D momentum-conserving fluids [38]. This is the anomalous heat conduction behavior dominated by a hydrodynamic effect, which is well known in non-equilibrium heat transport.

Altogether, this can be understood as a crossover from ballistic, to diffusive (kinetic), and then to anomalous behavior controlled by corresponding timescales, as can be seen in [23,39,40,41,42,43,44].

**Figure 3:**
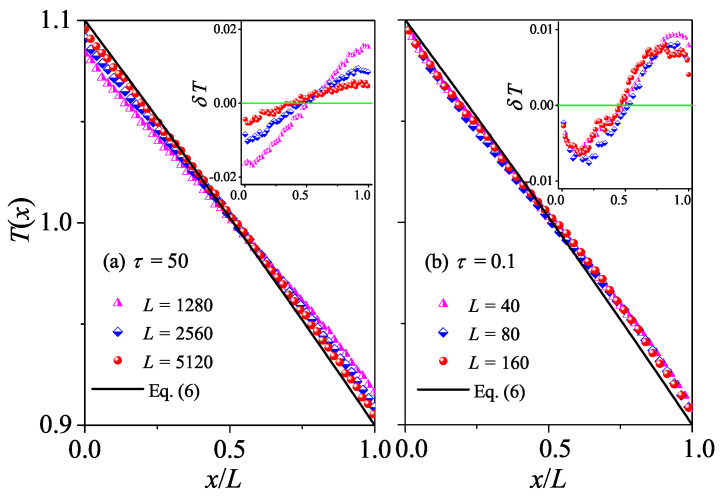
(Color online) Plot of temperature profiles T(x) for the 1D fluid system with different *L* values. Here, our numerical results are compared with the analytical Equation (6). In (**a**,**b**) we fix τ=50 and τ=0.1, respectively. Inset: Plot of the differences δT between the data and the black line, and the green line at δT=0 are for reference.

The difference between normal and abnormal heat conduction behaviors can be further appreciated also in the steady-state kinetic temperature profiles T(x). For systems with normal heat conduction, T(x) is predicted by Equation (6). In Figure 3a, this prediction is compared with our simulation results for τ=50. It is seen that there is good agreement between the results of our numerical simulations and Equation (6). To better appreciate the deviations from the prediction, we also plot the differences δT between the data and the black line. It is shown in the inset of Figure 3a that |δT| decreases with increasing *L*, as expected since |∇T|=ΔT/L decreases when *L* increases, indicating that the linear response can correctly describe the transport properties of the system for a long enough system. As introduced in the Section 2, for systems with abnormal heat conduction, the typical feature of the temperature profile is that T(x) is concave upwards in part of the system and concave downwards elsewhere, and this is true even for small temperature differences [29,30]. This is confirmed in Figure 3b by our numerical simulations for τ=0.1. Note that the data for three different *L* overlap with each other, implying that the deviations from Fourier’s behavior are not finite-size effects. Altogether, those numerical results again support our findings based on the length-dependence of the thermal conductivity, that heat conduction in the weak interactions is normal, while in the stronger interactions, it is abnormal.

### 3.2. Equilibrium Results

**Figure 4:**
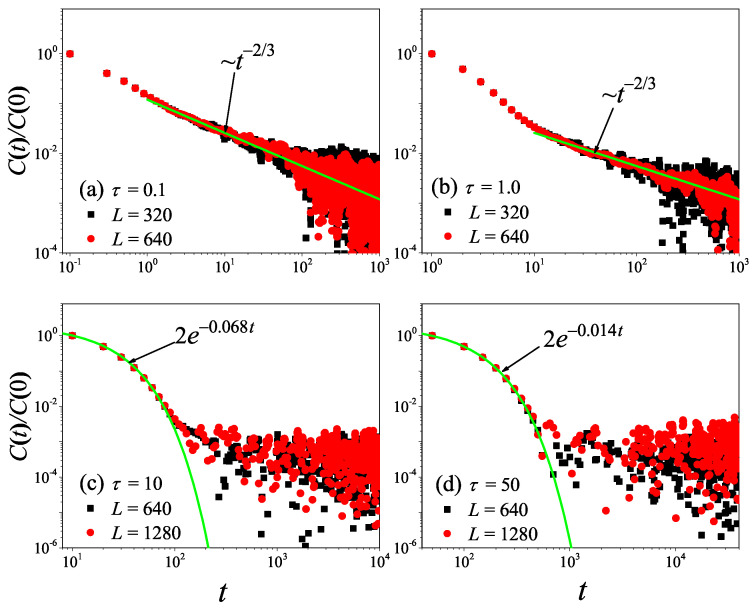
(Color online) Correlation functions $C\left(t\right)$ of the total heat current for the 1D fluid system with different τ values. For reference, the green solid line is the best fitting function for the data.

To check the results obtained in the 1D non-equilibrium modeling, we now turn to the comparison with the results obtained by the Green–Kubo formula in the equilibrium modeling. The results for the current time-correlation functions C(t) with different τ values are presented in Figure 4. It can be seen from Figure 4a,b that, for τ=0.1 and τ=1, the correlation function eventually attains a power-law decay $C\left(t\right)∼t^{\gamma }$ with γ=−2/3, fully compatible with the theoretical prediction of the 1D case [45,46]. Substituting it in Equation (7), and cutting off the integration as explained above, one obtains the superdiffusive scaling $\kappa_{\mathrm{GK}}∼L^{1/3}$, in agreement with our non-equilibrium modeling.

**Figure 5:**
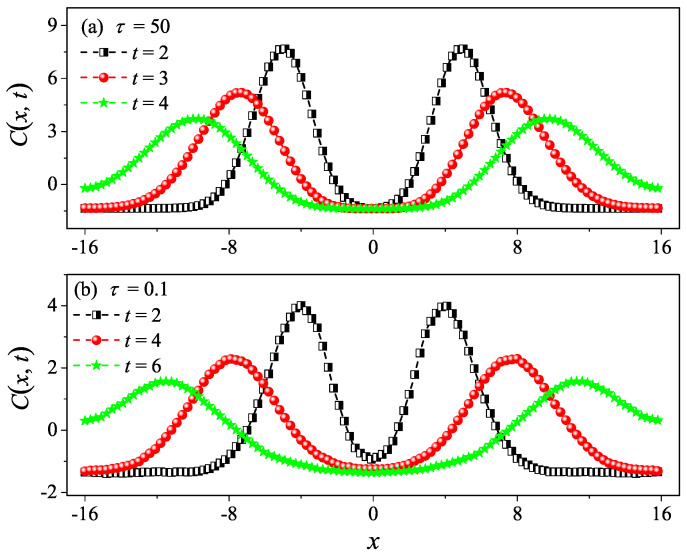
Numerical calculation of the spatiotemporal correlation function $C\left(x,t\right)$ for the 1D fluid system with τ=50 (**a**) and τ=0.1 (**b**). Here, the system length is set to be L=32. One can clearly see the two peaks (the hydrodynamic mode of sound) moving in the opposite direction away from x=0 in (**a**,**b**).

However, it is clear in Figure 4c,d that, for τ=10 and τ=50, C(t) undergoes a rapid decay at short times, and eventually, it begins to oscillate around zero (the negative values of C(t) are not shown in this log–log scale). The fitting function with the green solid line exhibits an exponential decay. To compare $\kappa_{\mathrm{GK}}$ and κ more accurately, we compute the sound speed $v_{s}$ of the system with the help of the spatiotemporal correlation function $C\left(x,t\right)$ of local heat currents defined in Equation (8). In Figure 5, we present $C\left(x,t\right)$ for the system size L=32. The two peaks representing the sound mode can be clearly identified in Figure 5a,b. Their moving speed $v_{s}$ is measured to be $v_{s}≃2.40$ for τ=50 and $v_{s}≃1.875$ for τ=0.1. In Figure 2, the horizontal line for τ=50 is obtained, truncating the integral in Equation (7) up to $L/v_{s}$ with $v_{s}=2.40$. It can be seen that $\kappa_{\mathrm{GK}}\left(L\right)$ agrees with κ. Thus, the equilibrium simulations are fully consistent with the non-equilibrium simulations.

## 4. Two-Dimensional Fluid

### 4.1. Non-Equilibrium Results

**Figure 6:**
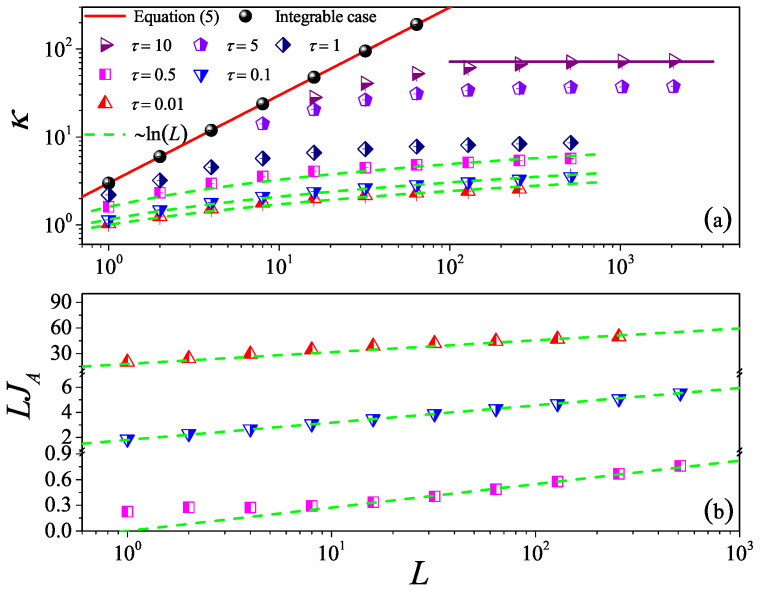
(Color online) (**a**) The thermal conductivity κ as a function of the system length *L* for the 2D square fluid system with different τ values. The symbols are for the numerical results, and for reference, the green dashed line indicates the divergence with *L* as ∼ln(L). For τ=10, the horizontal line denotes the saturation value of $\kappa_{\mathrm{GK}}$ obtained by Equation (7), where the integration is up a time $L/v_{s}$ with $v_{s}$ measured in Figure 9 bellow. (**b**) The log-linear scale is plotted to appreciate the relationship between the product of the anomalous flux $J_{A}$ and *L* and *L*. Here, we set W=L.

As for the 1D case, let us first report the dependence of κ on *L* for different τ values, as can be seen Figure 6a. To provide a numerical check, we show that the non-interacting (integrable) expression Equation (5) (red solid line) very accurately matches the simulation data (black circles). For the interacting systems, it can be seen in Figure 6a that, for the weak interactions (τ=10,5), κ tends to saturate and becomes constant as *L* is increased. This indicates that the normal heat conduction behavior for the nearly integrable 2D fluid system is also dominated by the kinetic effect. However, as τ decreases, κ is no longer constant but diverges with *L*. In particular, for τ=0.1 and τ=0.01, κ eventually approaches the scaling κ∼ln(L) like that predicted in 2D momentum-conserving systems [47]. Therefore, the scenario is similar to the 1D case and can be described as a crossover from kinetic to hydrodynamic regimes.

In order to demonstrate the crossover behavior, we assume that the flux can be decomposed as the sum into normal and anomalous contributions [41]

(9) $$J=J_{N}+J_{A},$$

From kinetic theory, we expect that $J_{N}\left(L\right)=a/\left(b+L\right)$ so we can use the conductivity data for large τ to estimate $J_{N}$ and thus deduce $J_{A}=J-J_{N}$. In Figure 6b, we show that, for the strong interactions (τ<0.5), $J_{A}$ is indeed proportional to log(L)/L. To our knowledge, this provides some of most convincing numerical evidence of the logarithmic divergence of the conductivity in 2D.

**Figure 7:**
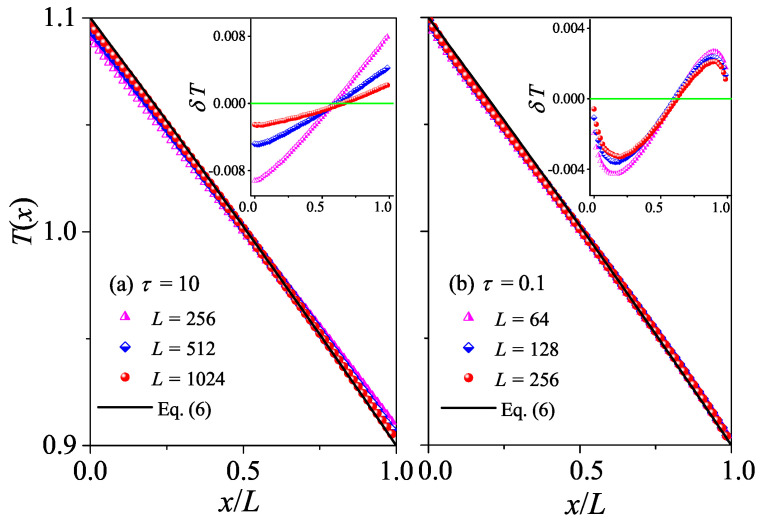
(Color online) Plot of the temperature profiles T(x) for the 2D square fluid system with different *L* values. Here, our numerical results are compared with the analytical Equation (6). In (**a**,**b**), we fix τ=10 and τ=0.1, respectively. Inset: Plot of the differences δT between the data and the black line, and the green line at δT=0 is for reference.

The difference between normal and abnormal heat conduction behaviors can be further verified by T(x) as in the 1D case. For systems with normal heat conduction, T(x) is predicted by Equation (6). In Figure 7a, this prediction is compared with our simulation results for τ=10. As expected, there is a good agreement between the results of our numerical simulations and Equation (6). However, for τ=10, T(x) is concave downwards in the left part of the system and concave upwards in the right part of the system. This again conforms to the temperature distribution characteristics of abnormal heat conduction.

### 4.2. Equilibrium Results

**Figure 8:**
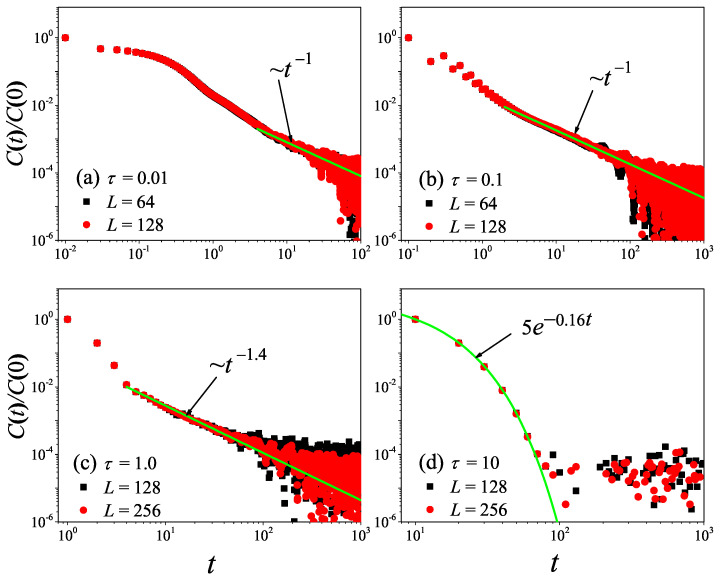
(Color online) Correlation functions $C\left(t\right)$ of the total heat current for the 2D square fluid system with different τ values. For reference, the green solid line is the best fitting function for the data. Here, we set W=L.

To check the results obtained above, we now turn to the comparison with the results obtained by the Green–Kubo formula in equilibrium modeling. The results for C(t) with different τ values are presented in Figure 8. It can be seen from Figure 8a,b that, for τ=0.01 and τ=0.1, the correlation function eventually attains a power-law decay $C\left(t\right)∼t^{\gamma }$ with γ=−1, fully compatible with the theoretical prediction of the 2D case [47]. Taking it in Equation (7), one will obtain the superdiffusive heat transport $\kappa_{\mathrm{GK}}∼\ln\left(L\right)$, in agreement with non-equilibrium data. Moreover, we note that the numerical result of τ=0.1 is in better agreement with the prediction than that of τ=0.01. This might be because, when τ=0.01, the collisions of randomly shifting cells should be taken to keep the Galilean invariance of the stochastic rotation dynamics [37].

However, it is clear in Figure 8 that, as τ further increases from τ=0.1 to τ=10, C(t) will change from power-law decay to exponential decay. This means that, as τ increases, the kinetic effects will play a dominant role, and thus heat conduction will change from anomalous behavior to normal behavior, as observed in Figure 6.

**Figure 9:**
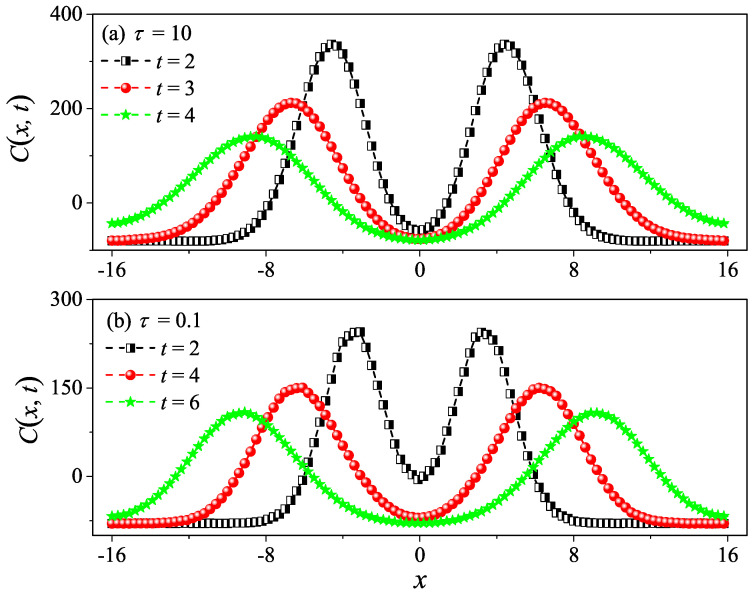
Numerical calculation of the spatiotemporal correlation function $C\left(x,t\right)$ for the 2D square fluid system with τ=10 (**a**) and τ=0.1 (**b**). Here, we set W=L=32. One can clearly see the two peaks (the hydrodynamic mode of sound) moving in the opposite direction away from x=0 in (**a**,**b**).

To compare $\kappa_{\mathrm{GK}}$ and κ more accurately, we compute the sound speed $v_{s}$ of the system as performed in the 1D case. In Figure 9, we also present $C\left(x,t\right)$ for the system size L=32. The two peaks representing the sound mode can be clearly identified in Figure 9a,b. Their moving speed $v_{s}$ is measured to be $v_{s}≃2.05$ for τ=10 and $v_{s}≃1.50$ for τ=0.1. In Figure 6, the horizontal line for τ=10 is obtained by truncating the integral in Equation (7) up to $L/v_{s}$ with $v_{s}=2.05$. It can be seen that $\kappa_{\mathrm{GK}}\left(L\right)$ agrees with κ. Again, the equilibrium simulations are fully consistent with the non-equilibrium simulations.

## 5. Three-Dimensional Fluid

### 5.1. Non-Equilibrium Results

**Figure 10:**
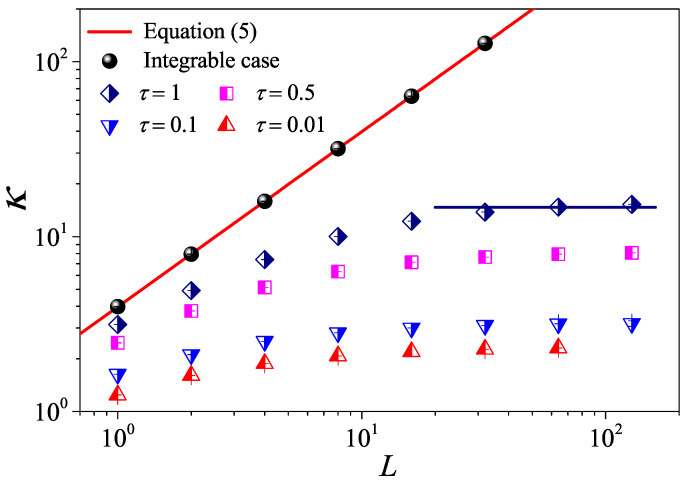
(Color online) The thermal conductivity κ as a function of the system length *L* for the 3D cubic fluid system with different τ values. For τ=1, the horizontal line denotes the saturation value of $\kappa_{\mathrm{GK}}$ obtained by Equation (7), where the integration is up a time $L/v_{s}$ with $v_{s}$ measured in Figure 13 bellow. Here, we set W=H=L.

To complete the study of dimensionality effects, we also performed a series of simulations for the 3D case. The results in Figure 10 demonstrate that, even here, the formula Equation (5) accounts very accurately for the non-interacting case (comparing the red solid line with black circles). The data for the interacting case (symbols in Figure 10) confirm that, even for the smallest τ considered, the conductivity converges to a finite value. As expected, Fourier’s law holds for a 3D fluid system with the momentum conservation.

**Figure 11:**
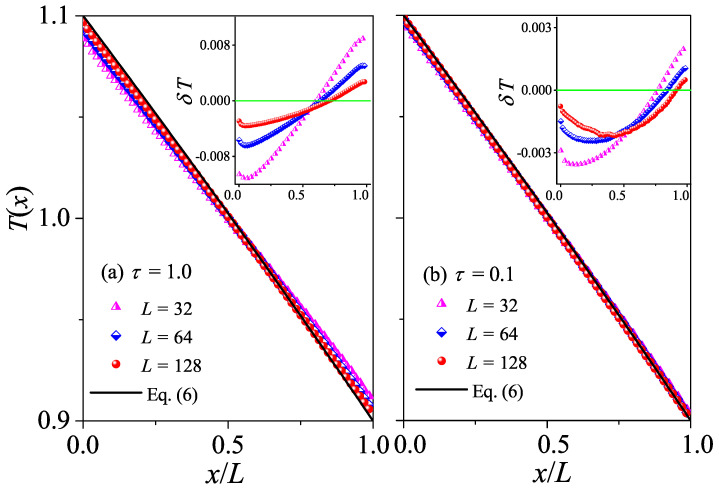
(Color online) Plot of the temperature profiles T(x) for the 3D cubic fluid system with different *L* values. Here, our numerical results are compared with the analytical Equation (6). In (**a**,**b**), we fix τ=1.0 and τ=0.1, respectively. Inset: Plot of the differences δT between the data and the black line, and the green line at δT=0 are for reference.

The normal heat conduction behavior can be further verified by T(x). For systems with normal heat conduction, T(x) is predicted by Equation (6). In Figure 11, this prediction is compared with our simulation results for τ=1 and τ=0.1. As expected, there is good agreement between the results of our numerical simulations and Equation (6), conforming to the temperature distribution characteristics of normal heat diffusion.

### 5.2. Equilibrium Results

**Figure 12:**
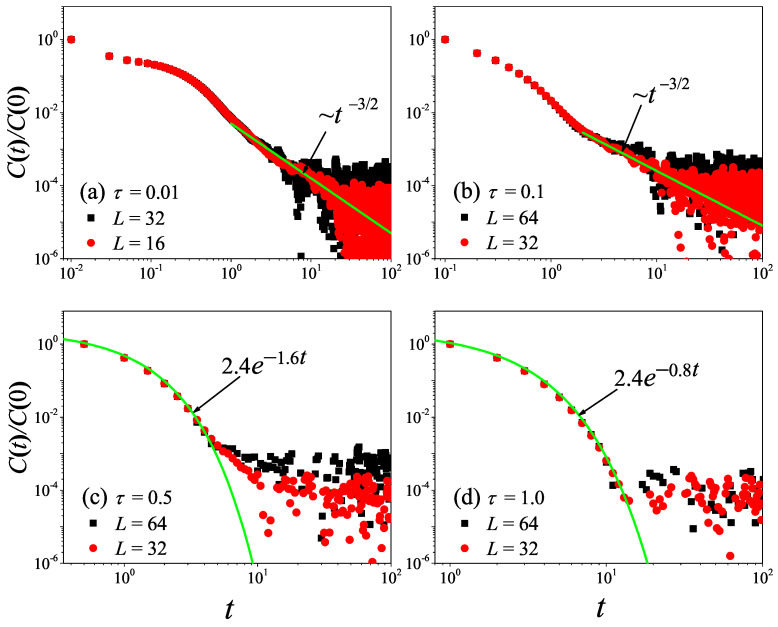
(Color online) Correlation functions $C\left(t\right)$ of the total heat current for the 3D cubic fluid system with different τ values. For reference, the green solid line is the best fitting function for the data. Here, we set W=H=L.

To check what was obtained in the 3D non-equilibrium modeling, we now turn to the comparison with the results obtained by the Green–Kubo formula in the equilibrium modeling. The results for C(t) with different τ values are presented in Figure 12. It can be seen from Figure 12a,b that, for τ=0.01 and τ=0.1, the correlation function eventually attains a power-law decay $C\left(t\right)∼t^{\gamma }$ with γ=−1.5, fully compatible with the theoretical prediction of the 3D case [47].

However, it is clear in Figure 12 that, as τ further increases from τ=0.01 to τ=1.0, C(t) will change from power-law decay to exponential decay. This means that, as τ increases, the normal heat conduction behavior observed in Figure 10 will change from being dominated by the hydrodynamic effect to being dominated by the kinetic effect.

**Figure 13:**
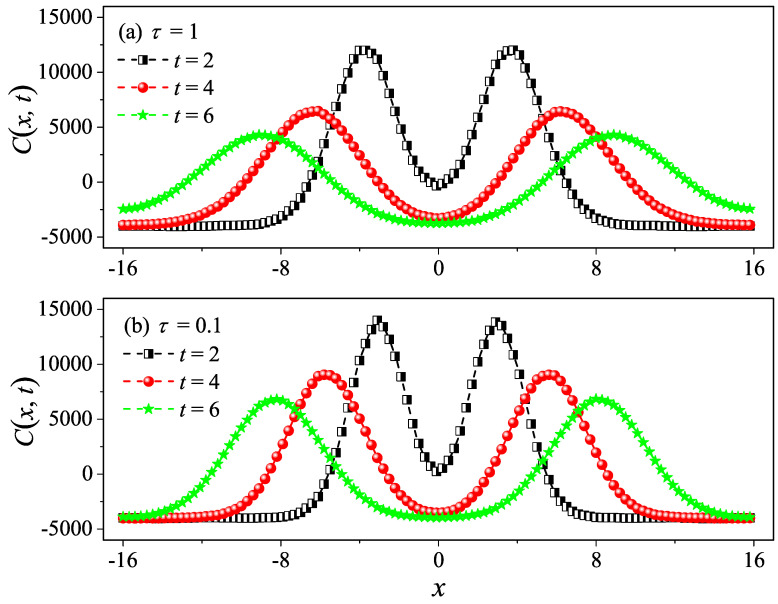
Numerical calculation of the spatiotemporal correlation function $C\left(x,t\right)$ for the 3D cubic fluid system with τ=1 (**a**) and τ=0.1 (**b**). Here, we set W=H=L=32. One can clearly see the two peaks (the hydrodynamic mode of sound) moving in the opposite direction away from x=0 in (**a**,**b**).

To compare $\kappa_{\mathrm{GK}}$ and κ more accurately, we compute the sound speed $v_{s}$ of the 3D system. In Figure 13, we also present $C\left(x,t\right)$ for the system size L=32. The two peaks representing the sound mode can be clearly identified in Figure 13a,b. Their moving speed $v_{s}$ is measured to be $v_{s}≃1.30$ for τ=1 and $v_{s}≃1.275$ for τ=0.1. In Figure 10, the horizontal line for τ=1 is obtained truncating the integral in Equation (7) up to $L/v_{s}$ with $v_{s}=1.30$. It can be seen that $\kappa_{\mathrm{GK}}\left(L\right)$ agrees with κ. Once again, the equilibrium simulations are fully consistent with the non-equilibrium simulations.

## 6. Dimensional Crossovers

Dimensional-crossover is a relevant topic for thermal transport in low-dimensional materials [15]. Indeed, in 2014, it has been experimentally observed in the suspended single-layer graphene [48]. In this experimental setup, the width of the samples is kept fixed and the thermal conductivity changes upon increasing their length is measured. As the length increases, it is natural to expect that a dimensional-crossover behavior from two dimensions to quasi-one dimension will occur. These research results have greatly enriched our understanding of heat conduction in lattice systems.

**Figure 14:**
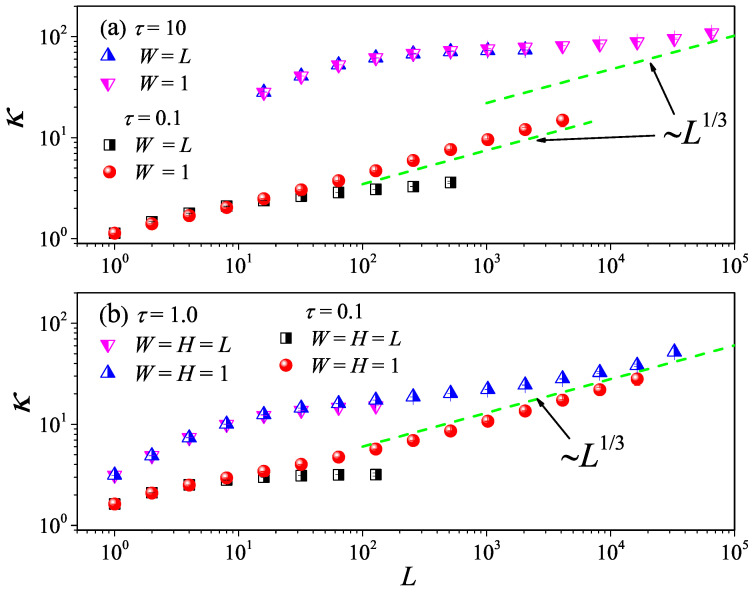
(Color online) The dimensional crossover behavior of heat conduction for the 2D (**a**) and 3D (**b**) fluid system with different τ values.

We show that the MPC approach can be successfully used to investigate this issue, considering 2D and 3D mesoscopic fluid models with fixed transverse sizes (W=1 in 2D and W=H=1 in 3D) and study how κ changes with *L*. It can be seen from Figure 14a that, in 2D fluid models, upon increasing the aspect ratio of the system, for both τ=10 and τ=0.1, κ eventually follows the 1D divergence law $\kappa ∼L^{1/3}$ as *L* increases. This means that, both in the cases dominated by the kinetic (τ=10) or hydrodynamic effect (τ=0.1), there exists a dimensional-crossover above a given aspect ratio. As shown in Figure 14b, in 3D fluid models, there is also a similar phenomenology. Altogether, these results confirm that the theories developed for the strictly 1D case effectively also extend quasi-1D, provided that the transverse extent of the sample is small enough.

**Figure 15:**
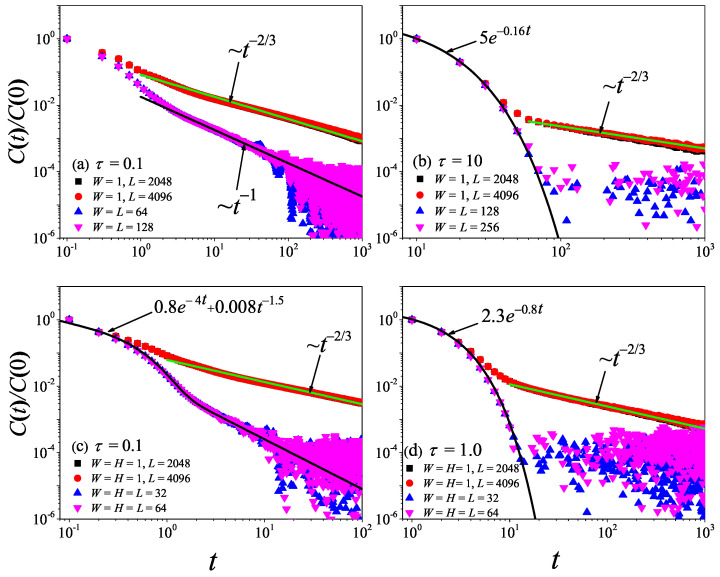
(Color online) Correlation functions $C\left(t\right)$ of the total heat current for the 2D and 3D fluid system with different τ values. For reference, the green and black solid lines are the best fitting functions for the data.

To further support the dimensional-crossover behavior of heat conduction observed above, we perform the equilibrium simulations of C(t) in 2D and 3D fluid models. The results for C(t) with different τ values are presented in Figure 15. We can see from Figure 15a,b that, in 2D fluid models, under the condition of increasing the aspect ratio of the system, for heat conduction dominated by the hydrodynamic effect (τ=0.1) or dominated by the kinetic effect (τ=10), C(t) will eventually change to a power-law decay $C\left(t\right)∼t^{-2/3}$. As shown in Figure 15c,d, in 3D fluid models, there is also a similar phenomenon that C(t) will eventually change to a power-law divergence for the hydrodynamic effect (τ=0.1) and the kinetic effect (τ=1).

## 7. Heat Transfer with Magnetic Field

Another issue that can be studied through the MPC dynamics concerns the influence of a magnetic field on transport [49,50,51,52]. It is generally believed that heat conduction behavior is normal in low-dimensional systems where momentum is not conserved [12]. However, there is a counterexample in a low-dimensional system with a magnetic field. Specifically, heat transport via the one-dimensional charged particle systems with transverse motions was studied in [49], where researchers studied two cases: case (I) with a uniform charge and case (II) with an alternate charge. An intriguing finding of this study is that, in both cases involving non-zero magnetic fields, the heat conduction behaviors exhibited anomalies, similarly to the case where momentum was conserved under the zero magnetic field condition. Remarkably, the abnormal behavior in case (I) is different from the case without the magnetic field, suggesting a novel dynamical universality class. Due to the presence of the magnetic field, the standard momentum conservation in such a system is no longer satisfied but is replaced by the pseudomomentum conservation [53]. Thus, there are two relevant questions: (1) Does the pseudomomentum conservation of a system lead to abnormal heat conduction? (2) Can the abnormal behaviors in both cases also be observed in low-dimensional fluids under the same pseudomomentum conservation?

**Figure 16:**
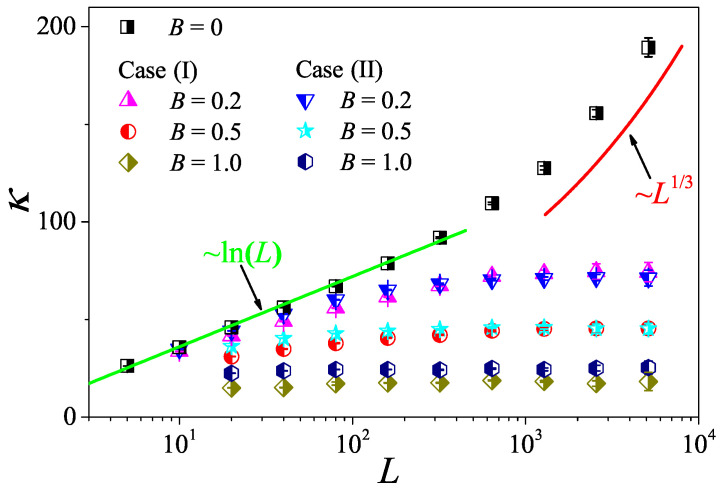
The heat conductivity κ as a function of the system length *L* for the 2D fluid system without and with a magnetic field. The symbols are for the numerical results. For reference, the green straight line is the best logarithmic fit, κ∼ln(L), and the red curve line indicates the divergence with *L* as $∼L^{1/3}$. Except for W=a=τ=0.1 and ρ=22, other parameters are consistent with those adopted in this paper.

The above two questions have only recently been well answered in our research [54], where it is shown that, under the same pseudomomentum conservation, the 2D fluid system with magnetic field can exhibit normal heat conduction behavior. Specifically, we consider a 2D system of charged particles as depicted in Figure 1b. In this system, a constant magnetic field perpendicular to the plane of motion, B=Bk, is imposed. The particles interact via the modified MPC dynamics to maintain the pseudomomentum conservation of the system (see [54] for details). To compare with the results obtained in [49], we also consider two cases: case (I) with uniform charges $e_{i}=1$ and case (II) with opposite charges on each half of particles, say $e_{i}={\left(-1\right)}^{i}$. In Figure 16, we plot the relation of κ vs. *L* for various *B* obtained by the non-equilibrium thermal-wall method. It is shown that, for B=0, the system with momentum conservation exhibits the crossover from the 2D to 1D behavior of the thermal conductivity under the condition of increasing the aspect ratio of the system. However, for B≠0, heat conduction behaviors in both cases with pseudomomentum conservation are normal because, as *L* increases, κ approaches a finite value.

Obviously, our above results are at variance with the findings in [49]. There, heat conduction in the presence of pseudomomentum conservation in two cases are abnormal. This observation, together with our results, thus clarifies that pseudomomentum conservation is not related to the normal and anomalous behaviors of heat conduction and provides an example of the difference in heat conduction between fluids and lattices in the presence of the magnetic field condition.

For completeness, we also mention that the MPC scheme can be extended to the case of charged particles that yields a self-consistent electric field. This situation is relevant for plasma physics and can be treated by coupling the MPC dynamics with a Poisson solver (see [55] and the references therein for details). The effect of the electric field on heat transport can be studied by this method: simulations reveal that the field does not affect significantly the hydrodynamics of the model, at least for not excessively large amplitude fluctuations [55].

## 8. Conclusions

We presented a series of numerical simulations demonstrating how the MPC method can be effectively employed to study the dimensionality effects on heat transfer in a simple confined fluid. Non-equilibrium dynamics can be simulated efficiently with the thermal-wall modeling of external reservoir and the results agree very well with Green–Kubo linear response. The data are statistically very accurate and span over a considerable range of system sizes. The overall theoretical scenario is confirmed by the data. It should be noticed that most of the publications in this context refer to lattice systems [12,13], so our results represent a relevant extension to the case where particles are free to diffuse through the simulation box. This supports the general validity of low-dimensional hydrodynamic theories and of the tight connection with Kardar–Parisi–Zhang physics in transport problems [46].

Another relevant finding is that the crossover from diffusive to anomalous regimes, seen in quasi-integrable chains [41], extends to the somehow simpler case of fluids. In particular, the decomposition of the current Equation (9) is an effective and simple way to assess the divergence law when the interaction is relatively weak and the accessible range of sizes too limited (as frequently occurs in practice).

We also illustrated how the important issue of dimensional crossovers and the effect of an applied magnetic field can be studied relatively easily via the MPC dynamics. A further extension would be to introduce the effect of chemical baths, namely to account for the exchange of particles with the environment [6]. This would allow us to study the basic features of the coupled transport process but also design and conceive novel possible applications.

## Data Availability

The original contributions presented in this study are included in the article. Further inquiries can be directed to the corresponding author.
